# Comparative frequency of specified adverse events following Vero cell culture-derived Japanese encephalitis and Vi capsular polysaccharide typhoid vaccines in U.S. military personnel, July 2011–August 2019

**DOI:** 10.1016/j.vaccine.2023.01.061

**Published:** 2023-01-30

**Authors:** Srihari Seshadri, Stacey W. Martin, Susan L. Hills, Limone C. Collins

**Affiliations:** a Immunization Healthcare Division, Public Health Directorate, Defense Health Agency, 7700 Arlington Blvd., Falls Church, VA 22042, USA; b Division of Vector-Borne Diseases, Arboviral Diseases Branch, Centers for Disease Control and Prevention, 3156 Rampart Rd, Fort Collins, CO 80521, USA

**Keywords:** Japanese encephalitis, Japanese encephalitis vaccine, Vero cell-derived, Adverse event, Retrospective, Vaccine safety, Delayed Hypersensitivity, Convulsions, Typhoid vaccine

## Abstract

Vero cell culture-derived Japanese encephalitis (JE) vaccine (JE-VC; Ixiaro) was approved in the United States in 2009. The previous JE vaccine, an inactivated mouse brain-derived vaccine, had been associated with rare, but serious, allergic and neurologic adverse events (AE). Studies and AE surveillance have supported JE-VC’s safety, but one evaluation among military personnel found elevated hypersensitivity and neurologic AE rates. However, co-administration of multiple vaccines to some personnel might have affected results. We retrospectively compared rates of hypersensitivity and neurologic AEs within 28 days following vaccination of military personnel with JE-VC or parenteral Vi capsular polysaccharide typhoid vaccine administered without other vaccines from July 1, 2011, through August 31, 2019. Rates of most events were similar between the vaccines. Only delayed hypersensitivity reactions occurred more frequently following JE-VC (rate ratio: 4.2, 95 % CI 1.2–15.3; p = 0.03), but rates were low for both vaccines. These results support JE-VC’s safety.

## 1. Introduction

Japanese encephalitis (JE) virus, a single-stranded RNA flavivirus, is a leading cause of encephalitis in Asia and the western Pacific [1]. JE virus is transmitted through the bites of infected mosquitoes, primarily *Culex* species. While most JE virus infections are asymptomatic, encephalitis can develop in < 1 % of persons and is often severe. Initial symptoms can include sudden onset of head-ache, vomiting, and fever, followed by mental status changes, weakness, convulsions, focal neurologic deficits, and movement disorders. Of those who develop encephalitis, 20 %–30 % die, and 30 %–50 % of survivors experience serious neurologic, cognitive, or psychiatric sequelae. There is no specific treatment for JE. U.S. persons who travel abroad to endemic areas are at risk of JE virus infection, but preventive measures such as mosquito avoidance and vaccination can reduce the risk [2].

In March 2009, the U.S. Food and Drug Administration (FDA) licensed Vero cell culture-derived JE vaccine (JE-VC; Ixiaro, manufactured by Valneva Austria GmbH) for use in persons aged ≥ 17 years. In 2013, licensure was extended to include children aged ≥ 2 months. As JE-VC became available in the United States, the production of mouse brain-derived JE vaccine (JE-MB; JE-VAX) was discontinued, and all doses expired by May 2011. Although JE-MB had been administered safely and offered protection for hundreds of thousands of persons who received the vaccine, it was noted to be associated with rare, but serious, allergic and neurologic adverse events [3].

In pre-licensure clinical trials among approximately 5,000 adults, rates of local and systemic adverse events following JE-VC were comparable to rates among recipients of placebo adjuvant [2]. However, given the limited safety data available at the time of initial licensure, the FDA required the manufacturer to conduct a post-licensure study to evaluate the safety of JE-VC in a large population [4]. Since most JE-VC doses used in the United States are administered to military personnel, the manufacturer conducted the study using the Defense Medical Surveillance System (DMSS) data [5]. The DMSS is a relational database that receives U.S. military service members’ health information from multiple sources, and contains data including medical events, diagnoses, and vaccinations [6,7].

The manufacturer’s analysis showed that among 21,347 U.S. military personnel vaccinated with JE-VC from July 2010 through May 2011, there were no statistically significant increased incidence rates for multiple neurologic and hypersensitivity events compared with the rates among personnel vaccinated with JE-MB [5]. However, some potentially serious adverse events, including convulsions and delayed hypersensitivity reactions, were reported at rates of ≥ 1 case per 1,000 person-years. Because the assessment was conducted among military personnel who regularly receive multiple vaccines at a single visit, including vaccines infrequently used among the civilian population (e.g., smallpox, anthrax), the impact of coadministration of other vaccines on the adverse event rates was unclear. Other vaccine adverse event data gathered since licensure such as data from passive adverse event surveillance systems are also limited by frequent concomitant administration of other vaccines.

Given the findings of the military study and the value of additional data given concerns about similar events with the previous mouse-brain derived vaccine, we conducted a study to assess selected neurologic and hypersensitivity adverse events in military personnel following JE-VC and a comparator vaccine after removing potential confounding caused by coadministration or recent receipt of other vaccinations.

## 2. Methods

Data from military personnel vaccinated with JE-VC were compared to those from vaccinees who received parenteral Vi capsular polysaccharide typhoid vaccine (TY-VC; Typhim Vi, manufactured by Sanofi Pasteur). This typhoid vaccine has been used for approximately 30 years in the United States and has been demonstrated in studies and post-marketing surveillance to be well-tolerated and have a favorable safety profile [8–10]. Deidentified patient-level data were extracted from DMSS for all male and female active-duty U.S. military personnel ≥ 17 years of age who received JE-VC or TY-VC from July 1, 2011, through August 31, 2019, without receiving any other vaccinations in the 28-day period before or after the relevant vaccination. Data collected included demographic information, branch of military service, the occurrence of preselected hypersensitivity and neurologic adverse events of special interest (AESI), hospitalizations, and deaths in the 28-day period following vaccination. The AESI included six neurologic and four hypersensitivity events, extracted based on International Classification of Disease (ICD), 9th Revision, Clinical Modification (CM) and ICD-10-CM codes. (Table 1). An individual could be included more than once if multiple doses meeting the criteria above were administered.

We compared the occurrence of AESI, hospitalizations, and deaths in the 28-day period following vaccination among personnel receiving JE-VC to events among those receiving TY-VC. The rate ratio (RR) was estimated using modified Poisson regression with robust error variances. Analyses accounted for possible repeated measures on a person and were conducted using SAS version 9.4. Factors including gender, age, and branch of military service were initially included in the Poisson models but were removed due to model lack of convergence or non-significance. Only the first report of a specific AESI in the 28 days following vaccination in an individual was counted. For anaphylactic reactions, we ran a secondary analysis shortening the risk window to a 2-day period following vaccination.

The Walter Reed National Military Medical Center Institutional Review Board (IRB) deemed the protocol to be exempt from IRB review. The Centers for Disease Control and Prevention received a non-engagement determination. This research did not receive any specific grant from funding agencies in the public, commercial, or not-for-profit sectors.

## 3. Results

Data from a total of 718,239 military personnel were included. The median age of personnel was 25 years (interquartile range (IQR): 9) and the majority (87 %) were male (Table 2). Race and ethnicity of the included personnel were similar though the branch of service varied between personnel in the two vaccine groups. Overall, 1,029,055 doses of vaccine, 288,546 doses of JE-VC and 740,509 doses of TY-VC, were included. While the majority (69 %) of persons contributed a single eligible vaccine dose to the analysis, 22 % contributed two doses, and 9.1 % contribute three or more doses.

The combined rates of all selected hypersensitivity or neurologic AESI occurring in the 28 days following vaccination with JE-VC or TY-VC were similar (Table 3). The occurrence of anaphylactic reactions was similar between vaccines for both the 2-day and 28-day period following vaccination (data for the 2-day period not shown). Of the specific AESI, urticaria occurred most frequently with similar rates for both vaccines. The rates of the remaining nine AESI were low and similar between the vaccines; only delayed hypersensitivity reaction was found to be significantly associated with JE-VC vaccination (RR: 4.2, 95 % CI: 1.2–15.3; *p* = 0.03). Hospitalizations were rare and rates were not significantly different between the vaccines (RR: 0.7 95 % CI: 0.3–1.6; *p* = 0.35). Very few deaths occurred, and the rates were again similar in both groups (RR: 1.3 95 % CI: 0.2–7; *p* = 0.77).

## 4. Discussion

Our analysis of U.S. military personnel found an acceptable safety profile for JE-VC when compared to TY-VC. Overall, the rates of hypersensitivity or neurologic AESI, hospitalizations, and deaths following administration of JE-VC were comparable to those following TY-VC [8–10]. While we did detect a significant difference in the rates of delayed hypersensitivity with JE-VC compared to TY-VC, the rates for both vaccines were relatively low.

Hypersensitivity and neurologic adverse events were of interest when JE-VC was licensed because the previously available JE vaccine, JE-MB, had been associated with rare, serious allergic and neurologic adverse events [11]. Specifically, generalized urticaria and angioedema of the extremities, face, and oropharynx was of concern, with symptom onset sometimes delayed for up to 14 days after vaccine administration [11,12]. Severe hypersensitivity events following JE-MB were estimated to occur at rates ranging from 10 to 260 cases per 100,000 vaccinees [11,13]. Severe neurologic adverse events were also of concern, including rare cases of acute disseminated encephalomyelitis [14]. It was hypothesized that gelatin, used as a vaccine stabilizer, might have been responsible for the hypersensitivity adverse events, and the use of mouse brains as the substrate for virus growth might have contributed to the frequency of neurologic adverse events [15]. JE-VC does not contain a stabilizer and uses Vero cells as the substrate so it was expected to have a different and likely better safety profile. The similar rates and low case numbers of hypersensitivity and neurologic AESI in recipients of JE-VC and TY-VC in this analysis is reassuring.

Although we identified an elevated rate of delayed hypersensitivity events with JE-VC compared with TY-VC, the rate for JE-VC was substantially lower than in the previous military study that did not control for coadministration or recent receipt of other vaccinations [5]. In addition, convulsion rates were similar following JE-VC and TY-VC in this study, and again lower compared to the previous study. Finally, it is important to note that despite events occurring in temporal association with vaccination, this does not imply causality. Other large datasets analyzed after JE-VC’s licensure have not suggested any concerns with hypersensitivity, neurologic, or serious adverse events. Two analyses that reviewed adverse events reported to the U.S. Vaccine Adverse Event Reporting System (VAERS) in the 7 years after vaccine licensure, when > 1 million JE-VC doses were distributed, supported the vaccine’s favorable safety profile [16,17].

Our analysis had several limitations. The accuracy of classification of adverse events based on the ICD codes could not be assessed and might have impacted our estimates of adverse event rates. The specific aim of this study was to further investigate the occurrence of neurologic and hypersensitivity reactions in the same population from which the original AESI concerns were generated (i.e., active-duty military personnel) while removing potential confounding factors caused by coadministration of other vaccines. Given this, our findings are not generalizable beyond this population and the occurrence of these specific AESI could differ from the general population. DMSS data were deidentified so we could not conduct medical record review to enable an assessment of potential relatedness. Lastly, the DMSS may contain coding errors that misclassify the vaccines administered.

JE virus infection can cause severe clinical disease which often results in long-term sequelae or death. While the risk for JE is very low for most travelers to JE-endemic countries, some travelers will be at increased risk based on factors such as longer periods of travel, travel during the JE virus transmission season, spending time in rural areas, participating in extensive outdoor activities, and staying in accommodations without air conditioning, screens, or bed nets. All travelers to JE-endemic countries should take steps to avoid mosquito bites. Both travelers and their healthcare providers should consider the need for JE-VC vaccination. The findings from this analysis, in combination with results from prelicensure clinical trials and post-marketing surveillance, continue to demonstrate the good safety profile of JE-VC and provide reassuring data to support its use among at-risk travelers.

## Figures and Tables

**Table 1:** International Classification of Disease (ICD), 9th Revision, Clinical Modification (CM) and ICD-10-CM codes for selected adverse events of special interest (AESI).

AESI	ICD-9	ICD-10	ICD terminology
Hypersensitivity			
Anaphylactic reaction	999.4	T80.5	Anaphylactic reaction or shock due to serum
	995.0	T78.2	Anaphylactic shock or reaction, unspecified
		T88.6	Anaphylactic reaction due to adverse effect of correct drug or medicament properly administered
Angioneurotic edema	995.1	T78.3	Angioneurotic edema
Delayed hypersensitivity	999.52	T80.62	Other serum reaction due to vaccination
Urticaria	708.0	L50.0	Allergic urticaria
	708.1	L50.1	Idiopathic urticaria
	708.8	L50.8	Chronic urticaria or recurrent periodic urticaria
	708.9	L50.9	Urticaria unspecified
Neurologic			
Acute transverse myelitis	341.20	G37.3	Acute transverse myelitis in demyelinating disease of central nervous system
	341.21		
	341.22		
	356.4	G60.3	Idiopathic progressive polyneuropathy
Central nervous system inflammation and encephalopathy	323.01	G05.3	Encephalitis and encephalomyelitis in diseases classified elsewhere
	323.42	G05.4	Myelitis in diseases classified elsewhere
	323.51	G04.02	Postimmunization acute disseminated encephalitis, myelitis and encephalomyelitis
		G04.32	Postimmunization acute necrotizing hemorrhagic encephalopathy
	323.61	G04.00	Acute disseminated encephalitis and encephalomyelitis, unspecified
		G04.01	Postinfectious acute disseminated encephalitis and encephalomyelitis (Postinfections ADEM)
		G04.39	Other acute necrotizing hemorrhagic encephalopathy
	323.81	G04.81	Other causes of encephalitis and encephalomyelitis
	323.9	G04.90	Encephalitis and encephalomyelitis, unspecified
		G04.91	Myelitis, unspecified
	348.30	G93.40	Encephalopathy, unspecified
Convulsion	345.90	G40.909	Epilepsy unspecified, not intractable, without status epilepticus
	345.91	G40.919	Epilepsy, unspecified, intractable, without status epilepticus
	780.39	R56.9	Unspecified convulsions
Febrile convulsion	780.31	R56.00	Simple febrile convulsions
	780.32	R56.01	Atypical/Complex/Complicated febrile convulsions
Guillain-Barre Syndrome	357.0	G61.0	Acute Infective Polyneuritis/Post Infectious Polyneuritis/Guillain- Barré syndrome
Meningitis	322.0	G03.0	Nonpyogenic meningitis
	322.9	G03.9	Meningitis, unspecified

**Table 2:** Demographics and branch of military service of personnel receiving eligible doses of Vero cell culture-derived Japanese encephalitis vaccine (JE-VC) and Vi capsular polysaccharide typhoid vaccine (TY-VC), July 1, 2011–August 31, 2019.

Demographics	Overall N = 718,239	JE-VC* N = 210,571	TY-VC* N = 592,872
**Gender**			
Female	92,883 (12.9 %)	25,663 (12.9 %)	76,348 (12.9 %)
Male	625,355 (87.1 %)	184,908 (87.8 %)	516,523 (87.0 %)
Unknown	1 (0.0 %)	0 (0.0 %)	1 (0.0 %)
**Age at first included dose (years)**			
Median (IQR)	25 (9)	25 (9)	25 (9)
Range	17–68	17–65	17–68
**Race and ethnicity**			
Asian/Pacific Islander	33,215 (4.6 %)	11,067 (5.3 %)	27,298 (4.6 %)
Black	113,359 (15.8 %)	33,222 (15.8 %)	93,145 (15.7 %)
Hispanic	112,579 (15.7 %)	33,882 (16.1 %)	92,765 (15.7 %)
American Indian/Alaskan Native	8,833 (1.2 %)	2,052 (1.0 %)	7,783 (1.3 %)
White	403,000 (56.1 %)	116,101 (55.1 %)	332,833 (56.1 %)
Other	35,432 (4.9 %)	10,541 (5.0 %)	29,410 (5.0 %)
Unknown	11,821 (1.7 %)	3,706 (1.8 %)	9,638 (1.6 %)
**Branch of military Service**			
Army	229,360 (31.9 %)	71,121 (33.8 %)	182,742 (30.8 %)
Navy	224,606 (31.3 %)	41,441 (19.7 %)	205,309 (34.6 %)
Marine Corps	158,389 (22.1 %)	51,330 (24.4 %)	131,828 (22.2 %)
Air Force	101,040 (14.1 %)	46,630 (22.1 %)	68,178 (11.5 %)
Coast Guard	4,844 (0.7 %)	46 (0.0 %)	4,815 (0.8 %)

* The JE-VC and TY-VC columns are not mutually exclusive since personnel could contribute multiple eligible doses to the analysis. A person is only counted once within a column.

**Table 3:** Incidence and rate ratio (RR) for prespecified adverse events (AE) of special interest (AESI), hospitalizations, and deaths occurring in the 28-day period following vaccination among U.S. military personnel receiving Vero cell culture-derived Japanese encephalitis vaccine (JE-VC) or Vi capsular polysaccharide typhoid vaccine (TY-VC), July 1, 2011–August 31, 2019.

AESI	JE-VC N = 288,546 doses		TY-VC N = 740,509 doses		RR (95 % CI)	*p-value*
	n	AE rate per 100,000 doses	n	AE rate per 100,000 doses		
**Hypersensitivity AESI**						
Anaphylactic reaction	9	3.1	30	4.1	0.8 (0.4–1.7)	0.54
Angioneurotic edema	15	5.2	31	4.2	1.3 (0.7–2.3)	0.47
Delayed hypersensitivity	8	2.8	6	0.8	4.2 (1.2–15.3)	0.03*
Urticaria	150	52.0	342	46.2	1.1 (0.9–1.4)	0.23
Any hypersensitivity AESI	178	61.7	403	54.4	1.1 (1.0–1.4)	0.15
**Neurologic AESI**						
Acute transverse myelitis	0	0	4	0.5	NA	NA
Central nervous system inflammation and encephalopathy	0	0	6	0.8	NA	NA
Convulsion	25	8.7	71	9.6	0.9 (0.6–1.4)	0.72
Febrile Convulsion	0	0	1	0.1	NA	NA
Guillain-Barre Syndrome	0	0	0	0	NA	NA
Meningitis	5	1.7	11	1.5	1.2 (0.4–3.4)	0.77
Any neurologic AESI	30	10.4	91	12.3	0.9 (0.6–1.3)	0.44
**Hospitalization**	6	2.1	24	3.2	0.7 (0.3–1.6)	0.35
**Death**	2	0.7	4	0.5	1.3 (0.2–7.0)	0.77

* Statistically significant.

NA: Not applicable.

## Data Availability

The authors do not have permission to share data.
